# Analysis of Odorants in Marking Fluid of Siberian Tiger (*Panthera tigris altaica*) Using Simultaneous Sensory and Chemical Analysis with Headspace Solid-Phase Microextraction and Multidimensional Gas Chromatography-Mass Spectrometry-Olfactometry

**DOI:** 10.3390/molecules21070834

**Published:** 2016-06-25

**Authors:** Simone B. Soso, Jacek A. Koziel

**Affiliations:** 1 Environmental Science Interdepartmental Graduate Program, Iowa State University, 1201 Sukup Hall, Ames, IA 50011, USA; sbsoso@iastate.edu; 2 Department of Agricultural and Biosystems Engineering, Iowa State University, 4350 Elings Hall, Ames, IA 50011, USA

**Keywords:** semiochemicals, scent-markings, marking fluid, odor, *Panthera tigris altaica*, Siberian tiger, multidimensional GC-MS-olfactometry, 2-acetyl-1-pyrroline, solid-phase microextraction, volatile organic compounds

## Abstract

Scent-marking is the most effective method of communication in the presence or absence of a signaler. These complex mixtures result in a multifaceted interaction triggered by the sense of smell. The objective was to identify volatile organic compound (VOC) composition and odors emitted by total marking fluid (MF) associated with Siberian tigers (*Panthera tigris altaica*). Siberian tiger, an endangered species, was chosen because its MF had never been analyzed. Solid phase microextraction (SPME) for headspace volatile collection combined with multidimensional gas chromatography-mass spectrometry-olfactometry for simultaneous chemical and sensory analyses were used. Thirty-two VOCs emitted from MF were identified. 2-acetyl-1-pyrroline, the sole previously identified compound responsible for the “characteristic” odor of *P. tigris* MF, was identified along with two additional compounds confirmed with standards (urea, furfural) and four tentatively identified compounds (3-methylbutanamine, (*R*)-3-methylcyclopentanone, propanedioic acid, and 3-hydroxybutanal) as being responsible for the characteristic aroma of Siberian tiger MF. Simultaneous chemical and sensory analyses improved characterization of scent-markings and identified compounds not previously reported in MF of other tiger species. This research will assist animal ecologists, behaviorists, and zookeepers in understanding how scents from specific MF compounds impact tiger and wildlife communication and improve management practices related to animal behavior. Simultaneous chemical and sensory analyses is applicable to unlocking scent-marking information for other species.

## 1. Introduction

At the beginning of the 20th century there were over 100,000 tigers in the wild, which constituted nine *Panthera tigris* subspecies. Currently there are fewer than 3500 remaining in the wild [1] and about 7200 in captivity. This represents an approximate 97% decline since 1900. This reduction in population is primarily due to a plethora of anthropogenic factors including poaching, which has resulted in small effective population sizes and degradation of reproductive output; loss of habitat; decline in number of prey species; and climate change [1]. Recent estimates put the number of Siberian tiger population to be critically endangered, with approximately 350 remaining in the wild [1]. A worldwide scientific effort is required to prevent the complete eradication of the six remaining tiger subspecies (*Panthera tigris tigris*, *Panthera tigris corbeti*, *Panthera tigris jacksoni*, *Panthera tigris amoyensis*, *Panthera altaica*, and *Panthera tigris sumatrae*) [1,2].

Scent-marking is described as the most pervasive form of chemical signaling in mammals [3]. This complex mixture of numerous chemicals can result in a multifaceted interaction. Great cat markings have been studied, limitedly, to benefit conservation, specifically focusing on territoriality, dominance, and reproduction [4,5,6,7,8,9,10,11,12,13,14,15]. Researching these markings has led to a greater understanding of how great cats use scent markings: as a method for distinguishing amongst other conspecifics, neighbors, territorial boundary markings, and as behavioral and reproductive indicators [16,17].

Scent marking plays an integral role in animal identity. Scent marks have been used as key indicators of tiger population numbers and territorial distribution [14]. Previous research on *Panthera* has led to their species and sex identification from fecal and hair samples [18]. Scent-matching dogs used in the identification of tigers in the wild have proven to be 76% accurate [14]. This may be indicating that scent marks play a role in individuality and suggests that there is a strong association between characteristic odor and chemical composition of scent marks. Investigating scent marks could provide insight into the relationships between evolutionary changes and divergence across tiger subspecies which would assist with conservation and recovery efforts.

There has been limited research in the area of chemical and sensory analysis of great cat markings (Table 1). Scent marking has been analyzed in the African lion (*Panthera leo*), African cheetahs (*Acinonyx jubatus*), Indian leopards (*Panthera pardus fusca*), and puma (*Puma concolor*). Common procedures used to chemically characterize scent markings include: solvent-based extraction, headspace extraction, and solid-phase microextraction (SPME) for sample preparation and subsequent sample analyses using gas chromatography (GC), gas chromatography-mass spectrometry (GC-MS), liquid chromatography (LC), and thin layer chromatography (TLC) [4,7,8,9,10,11,19,20,21,22,23,24,25,26]. Over the last decade, GC-MS has been the leading analytical technology for scent mark characterization. 

Fifty-five volatile compounds were identified in lion urine through GC-MS analysis, but thirty-two were positively identified through chemical standard confirmation using multidimensional gas chromatography-mass spectrometry-olfactometry (mdGC-MS-O) [21,26]. The use of matrix assisted laser desorption ionization time of flight (MALDI-ToF) MS was useful to differentiate between the two compounds that migrated at nearly the same position in the gel electrophoresis used to identify cauxin in big cats [27].

The chemical composition of Siberian tiger (*Panthera tigris altaica*) MF has never been studied. To date, the MF composition of another species, *P. tigris tigris* (Bengal tiger) is by far best known. It is unique in that its chemical composition is very complex and it is the only subspecies of tiger MF ever to be studied for a comprehensive list of volatile organic compounds. Comparison of differences in the chemical composition and resulting odor of MF of subspecies of tigers has also never been conducted. Much of what is known about chemical composition of MF stems from chemical analyses [5,9,10,11,20]. The use of GC, GC-MS, and LC has enabled characterization of MF from Bengal tigers, specifically its lipid component [4,9,11,14,20]. Banks et al. [19] used GC analyses to identify trimethylamine, ammonia, methylamine, dimethylamine, 2-phenylethylamine, propylamine, triethylamine, and butane-1,4-diamine in Sumatran and Bengal tiger MF. Historically, confirmation of MF compounds identity has been attempted using GC column retention time [9]. However, this method of identification has its limitations and may be less accurate due to chemical co-elution in multifaceted scent-related matrices.

Poddar-Sarkar and Bramachary [20] utilized Bligh and Dyer’s [31] methanol-based solutions for the extraction of volatile compounds in Bengal tiger MF [8,20]. One hundred and fourteen volatile compounds (Table 1) have been identified in the MF of Bengal tigers [11]. With the exception of one study, Burger et al. [11], all previous tiger marking sample preparation techniques employed solvent-based extractions [4,9,20,28,29]. Burger et al. [11] used a “sample enrichment probe” (SEP) for the sample preparation of *P. tigris tigris* urine consisting of a short sleeve of 28 mg polydimethyl siloxane rubber affixed to a thin rod of an inert material [11].

Much thinner than the SEP, conventional SPME fibers consist of either a thin sorbent, polymer, or sorbent and polymer combined coating on a (e.g.,) fused silica glass fiber. This 1 or 2 cm fiber is attached to a ~200 µm o.d. inert wire supported inside a hollow needle. In comparison to commercial SPME fiber the volume of the coating and extraction surface area of a SEP PDMS rubber was likely larger, suggesting it has a superior extraction efficiency [10]. Besides the active compounds in MF, fixative lipids expelled with MF, assist in its long term persistence in the wild [20]. Thin layer chromatography determined that the lipid component constitutes 1.88 ± 0.75 mg/mL of MF and contains phospholipids, esters, free fatty acids, and glycerides [10,20].

Analytical techniques have unlocked a major purpose of scent marking, conspecific and interspecies communication [32]. Chemosensory analysis of scent markings has explained how they are vesicles which contain information that aids in the distinctions between animals of different sexes, ages, and social status and define the time during which a scent marking can be detected in tigers and other great cat species [6,12,20,21,28]. However, what an animal inhales and how it is processed has not been completely identified or understood [4,11,15,33].

2-Acetyl-1-pyrroline (2-AP) and phenylethylamine are the only compounds that have ever been associated with the characteristic odor resembling basmati rice, of Bengal tiger MF [4,28]. The methods for the identification of 2-AP aroma were based on simple yet robust human olfaction, which is limited in its ability to only detect odors at trace levels, e.g., 10^−7^ to 10^−11^ M in humans [34,35]. This method is also limited in identifying other compounds that may be contributing to the overall odor, so the improved sensory characterization with simultaneous chemical and sensory analyses can still be explored. The age of the sample and presumed loss of compounds over time can make it impossible to detect volatile compounds, specifically 2-AP using GC-MS [4]. The inability to identify 2-AP in Bengal tiger MF and urine was believed to be due to its rapid decay, and therefore limited period of odor identification [5]. Also, 2-AP is thought to be formed by a Maillard reaction during previous solvent-based sample preparation and not necessarily by natural occurrence [4,5,36]. 

Presently, no published research reports characterization of specific odorous chemical markers within scent marks to determine precisely which compounds are responsible for eliciting behaviors in tigers. Thus, there is a need to define characteristic odors by identifying key chemical constituents responsible for odor in a more reliable approach using analytical tools. Simultaneous chemical and sensory analysis is a powerful tool that could present a novel approach to odor characterization of MF of various mammals. The use of mdGC-MS-O could potentially define all odorous compounds and provide an improved library of odorous compounds contributing to eliciting behaviors and tiger identity. Multidimensional-GC-MS-O is a modern system that is utilized for the separation of volatile organic compounds (VOCs) and semi-VOCs. It utilizes multiple columns for the separation of polar and non-polar compounds and accounts for co-elution of compounds and chemical odors [37,38,39,40,41,42]. These are common problems associated with single column GC analyses [38,39]. Application of mdGC-MS interfaced with olfactometry (O) has the potential to accurately measure the influence of odor in scent marking detection in species that use chemical cues as their communication method.

Simultaneous chemical-sensory analyses have the potential to be more comprehensive, i.e., yielding valuable information about compound-scent links. In addition, methods based on mdGC-MS-O have very low method detection limits, e.g., 0.020 ng·L^−1^ to 0.022 ng·L^−1^ [40]. MdGC-MS-O has the capability, through its heart-cut mode, to improve the isolation and separation of complex mixtures, enhance odor characterization, and identify compounds [37,38]. Simultaneous chemical-sensory analysis has enabled the following findings: compounds responsible for the characteristic odor of live *H. axyridis* [37]; compounds contributing to the characteristic odor of livestock and poultry manure, rumen of beef cattle; linking specific odor with a volatile compound; the role of particulate matter as a carrier of odor; characterization of kairomones and characteristic odorants released by insects; and quantification of nutraceuticals in wine [37,38,39,40,41,42,43,44,45,46,47,48,49,50]. Application of mdGC-MS-O has the potential to measure the influence of odor in scent mark detection in species that use chemical cues as their communication method.

Solid phase microextraction (SPME) is particularly suited for characterization of volatiles from biological sources. SPME is a solventless extraction method that combines sampling and sample preparation. SPME fibers with assorted polymeric coatings can be either directly (e.g., by submersion in liquid) or indirectly (e.g., headspace) exposed to a sample. Different SPME coatings target specific categories of compounds based on their molecular weights, polarities, and functional groups. Volatiles and semi-VOCs passively diffuse onto the SPME fiber via adsorption, absorption or capillary condensation. SPME fiber coatings have a very high affinity for VOCs and semi-VOCs [45]. Thus, the sampling results in high pre-concentration and enrichment of compounds without the use of solvents and additional steps. There are relatively few publications that report the use of SPME for characterization of scent markings of large wild mammals [39,46,47,48]. SPME has been found to be better for the analysis of trace levels of analytes in the urine of Strepsirrhine families [51]. Automating headspace extraction with SPME was useful and a non-invasive method for monitoring reproductive status via the urine in elephants and other species [52,53,54]. 

The main objective of this study was to identify VOCs and odors of total MF associated with *P. tigris altaica* (Siberian tigers) with simultaneous chemical and sensory analyses using SPME and multidimensional GC-MS-olfactometry. Specifically, this study focused on: (1) Developing a sampling and analysis method for the identification of VOCs and semi-VOCs of Siberian tiger MF; (2) Determining which VOCs and semi-VOCs in Siberian MF are odorous and compare findings with literature; and (3) Developing an improved list of VOCs and semi-VOCs responsible for the characteristic aroma of tiger MF.

The use of SPME and mdGC-MS-O is a novel approach for improved characterization of odors of total tiger MF. The results of this study will: (a) aid in the development and improvement of semiochemical-based sample preparation and analytical techniques; (b) advance the understanding of the role of semiochemicals in other subspecies of tigers; (c) benefit the greater tiger worldwide population, in captivity and the wild; (d) determine the efficacy of mdGC-MS-O in the detection of 2-AP and other odor characteristic compounds; (e) determine the efficiency of SPME in extracting volatiles from MF of tigers; (f) potentially aid the rate of success in managing reproductive and social behaviors in a variety of species; and (g) improve semiochemical-based regulation of aggressive behaviors in animals; and (h) compare differences in the concentration, chemical composition, and odor of Siberian tiger MF in comparison to Bengal tigers. In the long-term, it may improve the chances of tiger survival. Investigating the MF of Siberian tigers could: provide insight into evolutionary modifications and/or adaptations, explain the importance of specific chemical compounds and their environmental persistence, and explain the role of these chemicals in species and gender differentiation and gender specific behavior.

## 2. Results and Discussion

### 2.1. Selection of Marking Fluid Extraction Parameters

**Figure 1:**
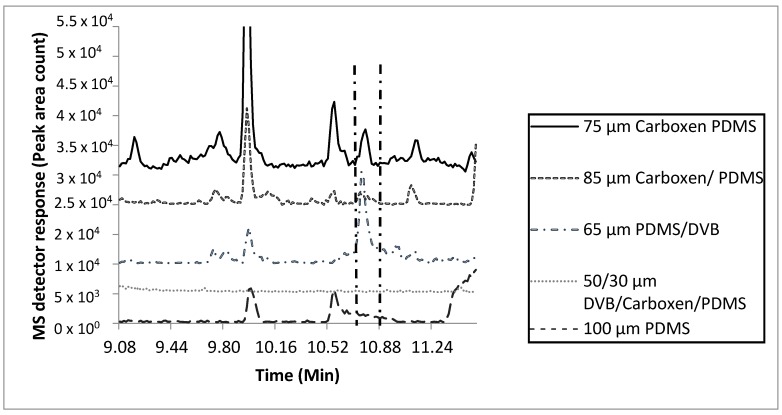
Effects of fiber coating type on SPME adsorption of 2-acetyl-1-pyrroline, the characteristic odorant compound released from marking fluid of *P. tigris altaica* with 85 µm CAR/PDMS, 50/30 µm DVB/CAR/PDMS, 100 µm PDMS, 65 µm PDMS/DVB, and 75 µm CAR/PDMS SPME fibers. Marking fluid (0.25 mL) and a stir bar were inserted into a 2 mL glass vial with a PTFE coated septa for a period of 30 min for equilibration. Samples (*n* = 3) were extracted at a temperature of 37 °C for 1 h. MS scan mode was total ion scan. Two min of the 40 min total scan is shown. Identification of 2-AP was accomplished with two fibers, the 75 µm CAR/PDMS and 65 µm PDMS/DVB SPME fibers. The 75 µm CAR/PDMS fiber had a peak area of 3.5 × 10^5^ counts and the 65 µm PDMS/DVB SPME fiber had a peak area of 8.9 × 10^5^ counts.

Extraction efficiencies using five fiber types (50/30 µm divinylbenzene/Carboxen/polydimethyl siloxane (DVB/CAR/PDMS), 85 µm Carboxen/PDMS (CAR/PDMS), 75 µm CAR/PDMS, 100 µm PDMS, and 65 µm PDMS/DVB, two temperatures (25 °C and 37 °C), two sample quantities (0.25 mL and 50 mL), and two extraction times (1 h and 24 h) were compared (Figure 1 and Figure S1–S3). Extraction parameters for the MF were based on the number of total and characteristic compounds detected, and peak area count comparisons of key compounds (Figures S1–S4). Based on these results, the 75 µm CAR/PDMS fiber with a 0.25 mL sample quantity, 24 h extraction at 37 °C was selected as the most efficient to characterize the VOCs within tiger MF. The 75 µm CAR/PDMS fiber was the only fiber coating that extracted enough mass for detecting the matching signature molecular ions and characteristic odors of all the “nutty” and “urinous” compounds emitted from tiger MF. Although the 65 µm PDMS/DVB SPME fiber was efficient at extracting enough mass for the detection and chromatographic identification of 2-AP, it was inefficient at the extraction of mass necessary for the detection of all 14 confirmed odorous compounds with a total of 32 odorous events detected with the 75 µm CAR/PDMS SPME fiber (Table S1, Figure S4, Table 2). Compared with the 75 µm CAR/PDMS SPME fiber, the 65 µm PDMS/DVB SPME fiber was only able to extract about half the number of compounds resulting in odorous events (18). In addition to 2-AP, the use of the 75 µm CAR/PDMS SPME fiber resulted in the identification of two (confirmed with chemical standards) compounds (urea, furfural), and four compounds tentatively identified as ((*R*)-3-methylbutanamine, 3-hydroxybutanal, propanedionic acid, and (*R*)-3-methylcyclopentanone)) responsible for characteristic odor in tiger MF (Figure S4, Table 2).

2-AP was used as a reference compound to measure changes in peak area counts between different sample volumes and SPME extraction times. There were no statistical differences in concentrations of 2-AP between the 0.25 mL and the 0.50 mL sample size and extraction times using the 75 µm CAR/PDMS (Figure S3). Due to the limited number of samples available, the 0.25 mL quantity was selected as the sample size for this study. The 24 h extraction time was selected because the number of detectable odorous compounds increased two-fold with the 23 h increase in extraction time (Figure S4).

### 2.2. Identification of Volatile Organic Compounds in P. tigris altaica Marking Fluid

Thirty-two compounds were identified by chemical standards (except for 2-AP), peak area, odor detection, retention time, spectral matches with top five ion relative intensities (Table 2). An additional 48 unconfirmed unidentified peaks were determined to be present within *P. tigris altaica* MF (Table S1). Identification of four of these peaks was attempted because they were characterized as having ‘nutty’, ‘urinous’, and/or ‘corn-like’ aromas by the odor panelists. These compounds (2-acetyl-1-pyrroline, (*R*)-3-methyIbutanamine, 3-hydroxybutanal, propanedionic acid, and (*R*)-3-methylcyclopentanone) were considered to be 4 of the 7 characteristic compounds tentatively identified through spectral match with top five ion relative intensities, odor panelists’ detection, and published odor descriptors. *P. tigris altaica* MF was comprised of nine chemical groups. These include ketones (9), aldehydes (5), amines (1), amides (1), alcohols (7), acids (2), phenols (1), sulfur-containing compounds (2), and nitrogen-containing compounds (4). All of these compounds were matched with an MS NIST spectral library match of 80% or higher and with olfactory detection by a trained panelist. 

Fourteen of the total compounds had human-detectible aromas that matched their published odor descriptors (Table 2). Those compounds with no detectable odors were identified through retention time, spectral match with top five ion matching, and chemical confirmation (Table 2). An additional set of 21 odor events were detected by panelists, but the identity of the compounds was not confirmed with chemical standards, due to feasibility. Four of the 21 odor events were comprised of odorous compounds with ‘characteristic’ aroma notes.

**Figure 2:**
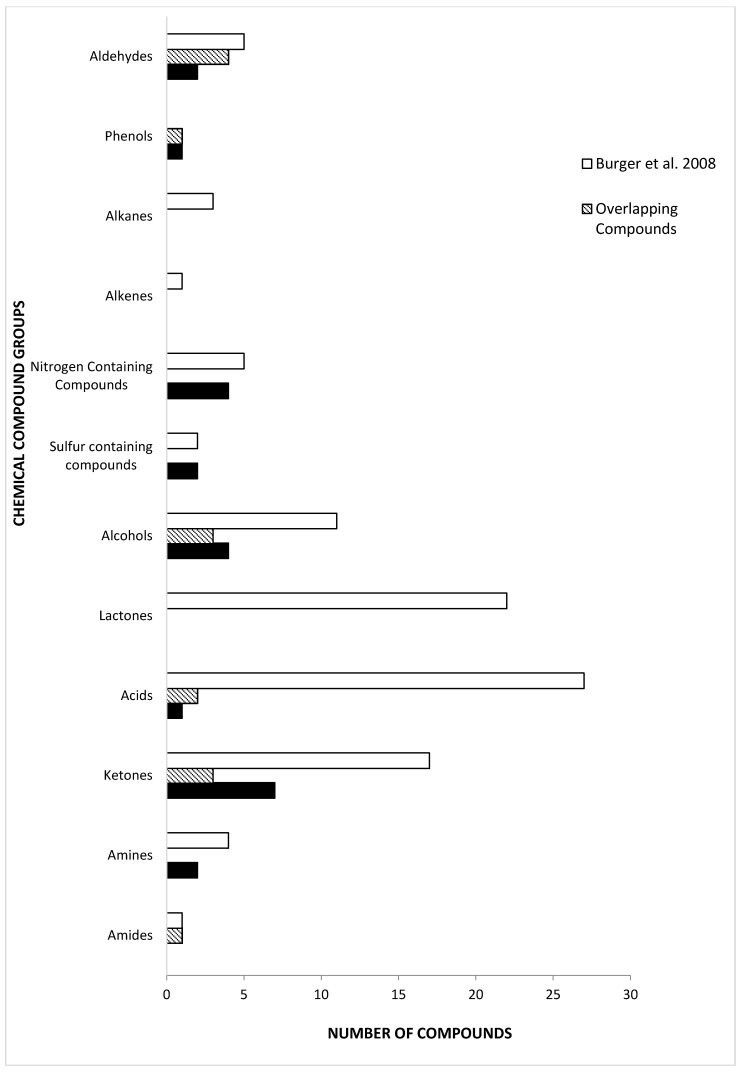
Comparison of chemical compound groups and number of identified with previously published *P. tigris tigris* urine and marking fluid compounds by Burger et al. [11].

There have been few reports published on chemical constituents of tiger MF. The majority of them focus on the Bengal tiger (*P. tigris tigris*) and the Sumatran tiger (*P. tigris sumatrae*) [5,11,14,20,65]. Previous studies on tiger MF have identified the constituents based on the analysis of separated MF into two separate fractions, the “lipid fixative” and “urine fraction” [11,20]. Burger et al. [11] is the only study published on Bengal tiger MF that analyzes both fractions, but separately. Compared to Burger et al. [11], the present study was able to detect equal number of sulfur-containing compounds in Siberian tiger MF (Figure 2). We also found five nitrogen-containing compounds in Siberian tiger MF, which is identical to the number previously determined in Bengal tigers [11]. Although the number of sulfur-containing compounds and nitrogen-containing compounds is the same, they were different in each subspecies. There were twice as many phenols in Siberian tiger MF than Bengal, but half of them were common to both. Aldehydes and ketones constitute similar numbers of compounds in tiger MF. Also, we determined the presence of 2-AP, previously undetected in Bengal tiger MF. The two groups with the highest number of common compounds were the alcohols and the aldehydes. Both studies identified 2-phenylethylamine as a constituent of tiger MF, albeit the identification in present study is preliminary (i.e., without chemical standard confirmation). 2-Phenylethylamine is found in the urine of carnivores and is one of the amine molecules that activates the trace amine-associated receptor in the epithelial tissue of the nasal cavity in bobcats and several other animals [66,67]. 2-Phenylethylamine is found in highest concentrations in the urine of tigers and lions [28,67]. Trimethylamine was identified and is a common compound identified in the MF of Bengal and Sumatran tigers, and African lions [19,20].

### 2.3. Odorous Volatile Organic Compound Detection

Addition of olfactometry to gas chromatography-mass spectrometry has enabled the detection of compounds in tiger MF that would otherwise not be identified. There were a total of 35 odors detected in Siberian tiger MF (Table 2 and Table S1). They ranged from “faint” to “intense” on the odor intensity scale (0%–100%). The overall characteristic scent of tiger MF can be characterized as “nutty” and “urinous.” Surrogate odor activity value (SOAV) measures the odor impact of a compound to the total odor of a sample. It is defined as the concentration (measured in chromatographic peak area count) of a single compound divided by the published odor detection threshold for that compound [68].

**Figure 3:**
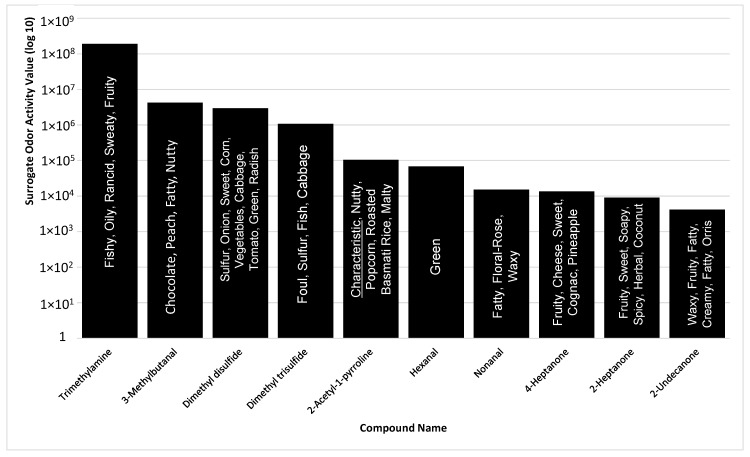
Summary of top 10 compounds, identified with standard chemical confirmation, in *P. tigris altaica* marking fluid with the highest surrogate odor activity values, SOAV (SOAV = odor detection threshold/peak area count) and their odor character descriptors. Confirmation of compounds was performed via chemical standards for all listed compounds with the exception of 2-AP.

**Figure 4:**
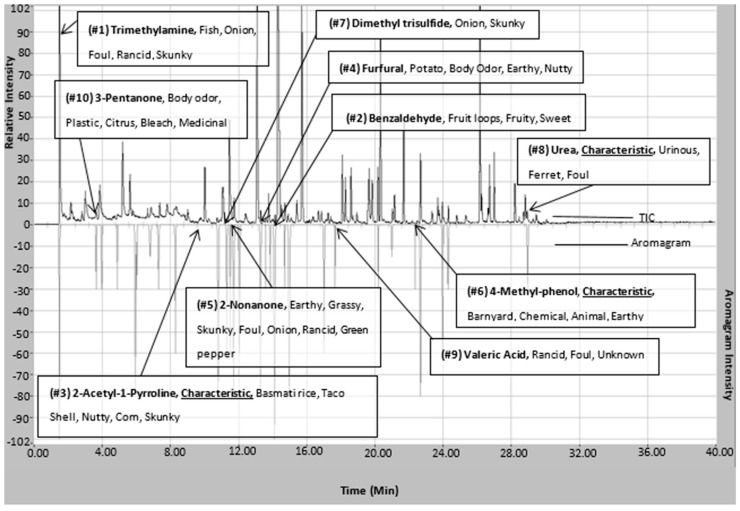
Chromatogram (top) and aromagram (bottom) resulting from simultaneous chemical and sensory analyses highlighting identified compounds in *P. tigris altaica* marking fluid responsible for the highest top 10 measured intense odors. Confirmation of compounds was performed via chemical standards for all listed compounds with the exception of 2-AP. 2-AP was confirmed using top five ions spectral match, retention time, and odor panelist observations. The odor characters listed are based on observed panelists’ evaluations.

Based on the compounds identified in the sample, the top ten SOAVs were trimethylamine, 3-methylbutanal, dimethyl disulfide, dimethyl trisulfide, 2-AP, hexanal, nonanal, 4-heptanone, 2-heptanone, and 2-undecanone (Figure 3). 2-AP, trimethylamine, and dimethyl trisulfide were the only compounds included in the top ten SOAVs that were organoleptically identified by panelists. The solitary use of SOAV for the determination of highly odorous compounds may not be inclusive of all highly odorous compounds being detected by animals. The determination of SOAVs is not applicable for compounds without published odor detection thresholds, leaving those compounds with potential odor influence unaccounted for. Organoleptic detection of scent-markings produces a list of odors that are detectable within the MF matrix. When determining the top ten most odorous compounds based on odor intensities selected by trained odor panelist, the list changes drastically (Figure 4).

Trimethylamine remains the highest ranked odorous compound. In addition, three of the seven compounds that are defined as being characteristic are also amongst the top ten odorous compounds in Siberian tiger MF. 2-AP is ranked 3rd in highest odor intensity among all of the odorous compounds in Siberian tiger MF. 2-AP is considered one of the main characteristic compounds associated with the “nutty” aroma of tiger MF [4]. The majority of the highly odorous compounds fall between the column retention time of 10 min and 17 min. This timeframe had the highest number of organoleptic identified peaks out of the 40 min chromatographic run. Urea and 4-methylphenol are two of the seven highly odorous characteristic compounds responsible for the urinous aroma of Siberian tiger MF. 4-Methylphenol, another characteristic compound, was ranked 6th in odor of highest odor ranking compounds. 4-Methylphenol is a highly odorous compound found in a variety of scent-markings of mammals including lions and swine [26,38]. This could explain its importance in intraspecies communication or evolutionary evolvement. In addition, Figure 4 illustrates the fact that “big peaks” do not necessarily result in detectable odor. Significant odors are sometimes causes by highly potent odorants represented by “small peaks”. This highlights the usefulness of simultaneous chemical and sensory analyses. 

### 2.4. Determination of Characteristic Compounds from P. tigris altaica Marking Fluid

Amongst the various odors that were observed, seven compounds were responsible for the key characteristic odor of Siberian tiger MF. These compounds include 2-AP, 3-methylbutanamine, (*R*)-3-methylcyclopentanone, propanedioic acid, urea, furfural, and 3-hydroxybutanal. The confirmation of these compounds was essential to prove their existence in tiger MF. The National Institute of Standards and Technology (NIST) mass spectral library was used to confirm the presence of the characteristic compounds along with odor confirmation (Figure S5). All of the spectral matches for these compounds were above 75%.

2-Acetyl-1-pyrroline is a compound previously identified as the characteristic compound of Bengal tiger MF [4,5,32]. The only method proven to identify this compound was paper chromatography and human organoleptics [4,5]. Burger et al. used SEP-GC-MS analysis and was unable to detect 2-AP [11]. The use of GC-MS-O allowed for a more precise and advanced identification of the 2-AP aroma area so that better software background removal could be done to match (84% spectral match) the compound. Upon refining the analytical technique using more sophisticated instrumentation with high sensitivity and odor capability, we were able to detect 2-AP, contrary to the previous review by Brahmachary and Poddar-Sarkar [5] (Figure S6).

In using SPME, the sample is not altered or subjected to solvent influence and alteration through sample preparation. We have determined that the presence of 2-AP is a natural occurrence and not the result of a Maillard reaction. Previously, the use of GC and GC-MS could not account for the presence of 2-AP in Bengal tiger urine and MF, however through the introduction of SPME-md-GC-MS-O, 2-AP was identified. An additional reason for the positive identification of 2-AP in Siberian tiger MF could be due to higher concentrations of this compound in Siberian tiger scent-markings. The absence of 2-AP in the lipid portion of *Panthera tigris tigris* MF may explain that it may reside solely in the urine, however looking at only the lipid fraction or the urinous fraction of MF may result in a lower number of VOCs.

**Figure 5:**
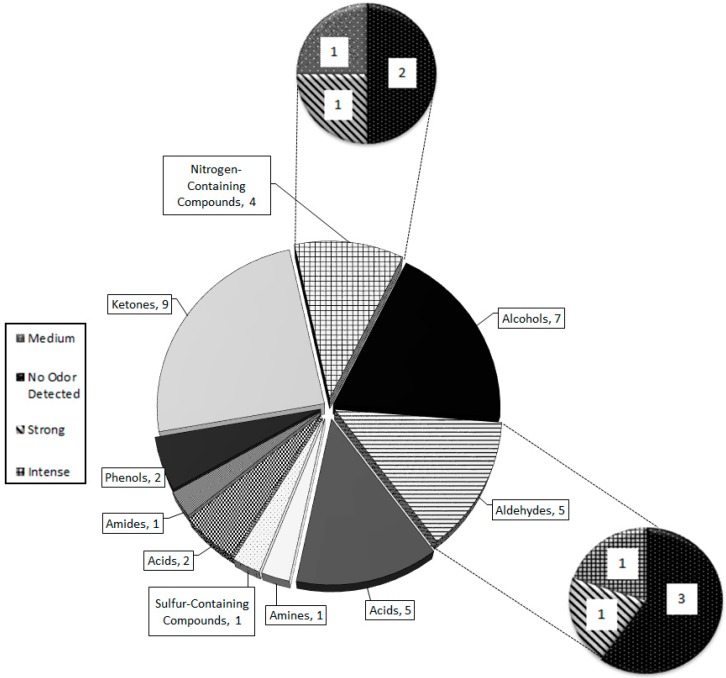
Total number of compounds responsible for the chemical and odor composition of *P. tigris altaica* marking fluid. The chemical groups having the highest number of intense, medium, and strong odor compounds were the ketones, aldehydes, acids, and nitrogen-containing compounds.

All of the characteristic compounds belong to one of five groups: amines, aldehydes, ketones, nitrogen-containing compounds, and acids (Figure 5). Ketones have the greatest number of odorous compounds with high intensities amongst all of the nine chemical groups that comprise tiger MF. Aldehydes (5) and nitrogen-containing compounds (4) had the largest number of medium-to-intense odorous compounds. Alcohols and amides had the highest number of undetectable odor compounds (Figure 5).

## 3. Materials and Methods

### 3.1. Standards and Solutions

The present study was carried out in the Atmospheric and Air Quality Laboratory of Iowa State University. Confirmation of the MF compounds was performed through identification with standards (if commercially available and feasible), GC column retention time, matching with Version 2.0 NIST Mass Spectral Search Program library, and matching of odor with odor data bases (e.g., Flavornet and Human Odor Space, The Good Scents Company, and Leffingwell & Associates). 

2,4,6-Trimethylpyridine can be used as an internal standard for the confirmation of 2-AP. Previous studies of Grimm et al. [69] and Ying et al. [70] used 2,4,6-trimethylpyridine as an internal standard for the quantitative and qualitative analyses of 2-AP in rice (*Oryza sativa L*.) [70] and additional aromatic rice and Panda (*P. amaryllifolius*) [69]. The conditions for analysis of 2-AP from *Oryza sativa L*. and *P. amaryllifolius* were optimized using HS-SPME/GC-FID and GC-MS. 20 mg of 2,4,6-trimethylpyridine and 20 µL of deionized water were inserted into a 22 mL vial at 80 °C for 30 min. One cm of the 50/30 DVB/CAR/PDMS fiber was exposed to this shaken vial to adsorb volatile compounds for 20 min [48,49,50]. 

### 3.2. Animal Subjects

We collected scent-marking samples from one male and one female adult Siberian tiger (*Panthera tigris altaica*) from the Blank Park Zoo. At the time of sampling, the female tiger was approximately 16 years old and the male was 19 years old. The animal subjects were fed and monitored daily by keepers and veterinary staff within the zoological grounds. Animals were cared for by the standards indicated by the Institutional Animal Care and Use Committee for Iowa State University and the Blank Park Zoo. No animals were harmed during the course of this study.

### 3.3. Marking Fluid Collection Processes

**Figure 6:**
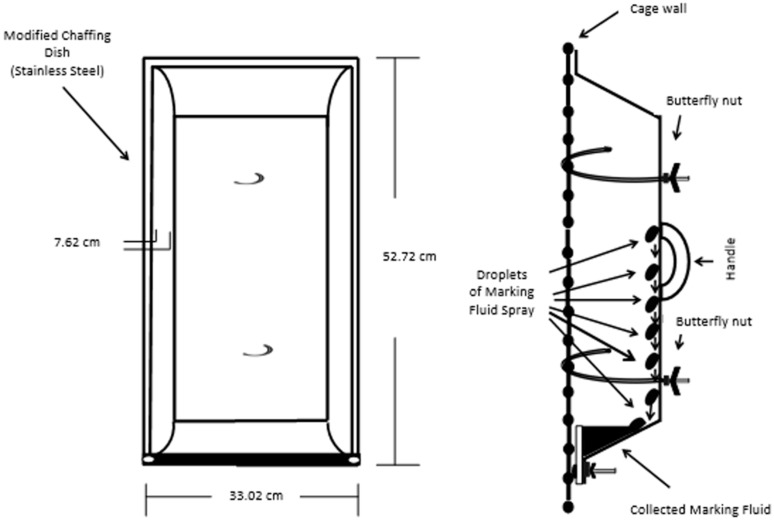
Marking fluid collection device and mode of collection.

The development of a sampling and analysis method for the identification of VOCs and semi-VOCs of Siberian tiger MF required the proper collection of samples. The indoor enclosures were used as the areas for collection. The floors and walls of the enclosures were power washed and scrubbed to reduce background in the sample. A 20 mL sample of the water used to wash the surfaces of the enclosure was collected to account for potential contamination. MF was collected using two different collection devices (e.g., collection trays and aluminum foil) (Figure S7). Four MF collection devices were hung varyingly on the portions of the caged wall of the indoor enclosure that are ≥0.90 m (≥3 ft) high (Figure 6, Figures S7 and S8) at the Blank Park Zoo. 

The wall behind the caged area was covered in aluminum foil to prevent the loss of MF sample. Separately, the animals were in the enclosure with the collection devices and allowed to roam freely between two enclosures simultaneously. Upon a marking event (Figure S9) the Pasteur pipettes were used to remove the MF from the collection devices (Figures S7 and S8) and the MF was pipetted into a 22 mL clear glass screw cap vials with a polytetrafluoroethylene (PTFE)-lined silicone septa vial that was properly labeled and stored in a portable cooler with ice packs. Approximately 80 mL of MF samples were collected. The collection process occurred over a 1-month period to reduce animal stress. After returning from the field, the samples were placed in a −20 °C freezer before analysis based on Burger et al. [11].

### 3.4. Sampling and Sample Preparation of Panthera tigris altaica Marking Fluid and Urine

Solid-phase microextraction method development was implemented to determine the most efficient parameters to extract the highest number of odorous volatile compounds. Five treatments (time-1 h and 24 h, sample size-0.25 mL and 0.50 mL, agitation method-static or magnetic stirring, and temperatures 25 °C and 37 °C) were applied to five SPME fiber coatings (85 and 75 µm CAR/PDMS, 50/30 µm DVB/CAR/PDMS, 100 µm PDMS, 65 PDMS/DVB). Fiber conditioning was based on manufacturer’s requirements. Fiber coating selection was based on the coating’s ability to attract and adhere to volatile and aromatic compounds previously identified in the chemical constituents of Bengal tiger MF and urine [42,43,44,45,46,47,48,49]. The experimental design is defined in Table 3. 

Prior to their use, all of the vials (2 mL Supelco^®^, Bellefonte, PA, USA), septa (polytetrafluoroethylene (PTFE)-lined silicone, Supelco^®^), and stir bars (0.20 cm × 0.50 cm, Fisher Scientific^®^, Rockville, MD, USA) were cleaned with sodium hydroxide (NaOH) and placed in the oven at 225 °C overnight to off-gas the impurities and prevent cross-contamination. For each experiment a defined quantity of sample was inserted into a 2 mL vial with a stir bar (agitation studies) or without one. These samples were kept in a −20 °C freezer until analyzed. Upon analysis, the sample was retrieved and brought to the desired temperature with a Fisher Scientific Isotemp Heated Magnetic Stirrer/Hotplate for a period of 30 min. For agitation studies, the magnetic stirrer was set to 1000 rpm for optimal vortical flow. This allows for the mass transfer of VOCs and semi-VOCs into the headspace. The selected fiber was inserted and pierced the septum remaining in a vertical position for the determined extraction period, removed immediately and manually injected into the GC injection port for analysis. Each experiment was replicated three times (*n* = 3) for each animal in the study. Each replicate used a separate 0.25 mL sample. 

### 3.5. Sample Analysis

Simultaneous chemical and sensory analyses of MF was performed using two modes (full Heart-cut and Selected Ion Monitoring) on a mdGC-MS-O instrument (Microanalytics, Round Rock, TX, USA). The MF was used to develop the SPME methodology for the analysis of *P. tigris altaica* MF. During SPME method development, the samples were run on the mdGC-MS-O in full Heart-cut mode (full HC). During this mode the heart-cut valve was open between 0.05 and 35 min run-time. The run parameters used were: injector, 240 °C; FID, 280 °C, column, 40 °C initial, 3 min hold, 7 °C·min^−1^, 240 °C final, 8.43 min hold; carrier gas, GC-grade He. The GC operated in a constant pressure mode, maintaining the mid-point pressure at 8.5 psi. During full HC mode, the midpoint heart-cut valve was opened for the pre-determined period that ranged the whole GC run (40 min) to allow transfer of compounds from column 1 to 2. This was controlled by the automation system MultiTrax^TM^ V. 6.50 (Microanalytics). Spectra were collected in three scan groups. Scan group 1 ran from 0 to 8 min collecting compounds with molecular weights ranging from 0–150 at 10.26 scans/sec. Scan group 2 ran from 8 to 20 min collecting compounds with molecular weights ranging from 150–280 at 5.53 scans/s. Scan group 3 ran from 20 to 40 min collecting compounds with molecular weights ranging from 280–350 at 4.43 scans/s. Selected ion monitoring (SIM) mode was utilized for the detection of 2-acetyl-1-pyrroline. SIM was run at 1.6 cycles/s. The mass channels were *m*/*z* = 111, 69, 43, 41, 42 for 2-AP. The end of column 2 (30.00 m, 0.53 mm, film thickness, 0.50 µm fused silica capillary column coated with polyethylene glycol, WAX; SGE BP20) was always splitting effluent to the sniff port and MS for simultaneous chemical and sensory analyses. The sniff port was turned to the “On” position to insure all odors eluting from column 1 ventured to column 2. The split ratio between the MS and the sniff port was 1:3. The sniff port temperature was set at 240 °C to eliminate condensation. Humidified air (99.997% purity, Praxair, Inc., Danbury, CT, USA) was delivered at 5.7 psi to maintain constant humidity for panelists’ mucous membranes. The tip of the sniff port had a custom panelist designed nose cone developed at Iowa State University. AromaTrax^TM^ V. 8 (Microanalytics) and ChemStation^TM^ (Agilent, Santa Clara, CA, USA) software programs were used for data acquisition (Figure S10). The aromagram was formed when an odor event occurred and was defined in an area of chromatographic separation. During the odor event panelists were responsible for recording the period in which the odor originates and ends, editable odor character descriptors, and perceived odor intensity. The aroma intensity was evaluated on a 0%–100% scale with 0% indicating no odor, 15% indicating a questionable odor, 30% indicating a faint odor, 60% indicating a medium odor, 80% indicating a strong odor, and 100% indicating an intense odor.

### 3.6. Determination of Chemical Composition and Odor of Siberian Tiger Marking Fluid

SPME fiber selection was based on its efficiency in the number of compounds detected, retention time (RT), total peak area counts using the ChemStation integration tool [54,55,56]; number of odors detected using AromaTrax^TM^ V. 8, Microanalytics^©^, Round Rock, TX, USA) tools and highest average odor intensities [56,71]; and detection of characteristic odorants resembling tiger MF aroma. To account for potential subjective bias, an odor panel (2 mdGC-MS-O experts) judged and compared odor character and intensity, but only one panelist was responsible for odor determination in the study. The data sets collected were analyzed using AromaTrax^TM^, Benchtop/PBM (Palisade Corp., Ithaca, NY, USA), Automated Mass-Spectral Deconvolution and Identification System (AMDIS), the NIST library (NIST, 2005), and MSD ChemStation (Agilent). Confirmation of the presence of these chemicals was based on the use of standard chemicals (when available), Flavornet and Human Odor database [57], MSDS data, THE LRI and Odour Database [71], and http://www.leffingwell.com confirmation, as well as panelist odor identification confirmation. Changes in the number of odorous compounds, retention time, integration (number of compounds), peak area counts (via ChemStation), changes in odor intensity and descriptors (via Aromatrax) were measured.

### 3.7. Isolation of Characteristic Odorants with GC-MS-O System

The use of multi-dimensional GC-MS-O allows for all compounds to be on the pre-column (column 1, non-polar) to be transferred to the analytical column (column 2, polar) for better separation. This resulted in the development of an improved list of chemicals responsible for the characteristic aroma of tiger MF. Compounds that were identified as having similar characteristic (nutty or urinous aromas) descriptors to that of the total aroma of MF were selected as compounds of interest in defining the characteristic aroma. These seven compounds were identified via olfaction and spectral confirmation. Multitrax (Microanalytics) software was used to control the timing of the valves in the GC-MS-O mode so that full HC mode could be run. 

## 4. Conclusions

Thirty-two compounds were identified in the MF of Siberian tigers through the development of a novel sample preparation and analysis technique. Fourteen of these were identified through olfactometry analysis. These compounds consisted of ketones, nitrogen-containing compounds, sulfur-containing compounds, alcohols, acids, aldehydes, phenols, amines, and amides. Panelists determined seven compounds as possessing the characteristic ‘nutty’, ‘urinous’, and/or ‘corn-like’ aroma of Siberian tiger MF. 2-Acetyl-1-pyrroline, 3-methylbutanamine, (*R*)-3-methylcyclopentanone, propanedioic acid, urea, furfural, and 3-hydroxybutanal were characterized as contributing to the overall characteristic odor of Siberian tiger marking fluid. Five of these compounds (2-Acetyl-1-pyrroline, (*R*)-3-methyIbutanamine, 3-hydroxybutanal, propanedionic acid, and (*R*)-3-methylcyclopentanone) were identified through spectral matches with the top five ions, odor panelists’ detection, and published odor descriptors. This study is the first to identify 2-AP through separation and spectral/sensory match on a mdGC-MS-O and extractions with SPME in tiger marking fluid. It is the first study to analyze tiger MF in its totality, giving rise to a new chemical previously unidentified in other tiger subspecies. Simultaneous chemical and sensory analysis made it possible to identify compounds that otherwise may have been overlooked and continued to be undetected. This research can lead to collaborations amongst various facilities and conservation parks. Knowledge gained from this work could proliferate the species and reduce the human-wildlife conflict occurring in various countries. The approach used on this research can be used as a model for aiding conservation of other globally endangered species.

## Figures and Tables

**Table 1:** Comparison of sample preparation, chemical, and sensory methods used to characterize scent markings, and the relationship between chemical constituents of marking fluid and urine of great cats.

Species	Reference	Type of Marking	Sample Preparation	Chemical Analyses	Sensory Analyses	Identified Compounds	Commonality in Composition of MF and Urine
*Panthera tigris tigris*	Poddar-Sarkar, M. and Brahmachary, R.L. [9]	Marking fluid	Solvent-based extraction	GC-FID, GC, TLC, GLC, PC	Not conducted	Free fatty acids	Not conducted
	Poddar-Sarkar, M. [20]	Marking fluid	Solvent-based extraction	GC-MS, GC-FID, GC, TLC, GLC, PC	Not conducted	Total lipid of MF consists of sterol ester, wax ester, triglyceride, free fatty acids, free sterol, diglyceride, and monoglyceride	Not conducted
	Brahmachary, R.L. and Dutta, J. [28]	Urine	Solvent-based extraction	PC	Orqanoleptic testing with human nose detection	2-Phenylethylamine defined as the characteristic odor compound and biochemical marker of urine	Not conducted
	Brahmachary, R.L. et al. [4]	Marking fluid	Solvent-based extraction	PC, GC	Orqanoleptic testing with human nose detection	2-Acetyl-1-pyrroline identified as characteristic compound of marking fluid	Not conducted
	Burger, B.V. et al. [11]	Marking fluid and urine	SEP	GC-MS	Not conducted	98 volatile compounds confirmed including ketones, fatty acids, lactones in MF	Major constituents of urine fraction and of the whole MF were ketones and nitrogen compounds; 2-Acetylpyrroline was not detected in urine or marking fluid; 48 common compounds between urine and MF; Variability in polarity and volatility of compounds identified in urine; MF contains seven times as many VOCs as urine
	Brahmachary [29]	Urine	Solvent-based extraction	TLC	Not conducted	Putrescine and cadaverine components of urine were identified, but later studies (Burger et al. [11] did not report them	Not conducted
*Panthera tigris sumatrae*; *Panthera tigris tigris*	Banks, G.L. et al. [19]	MF and anal sac secretion	Solvent-based extraction	GC	Not conducted	Trimethylamine, ammonia, methylamine, dimethylamine, 2-phenylethylamine, propylamine, triethylamine, and butane-1,4-diamine were found in Sumatran and Bengal tiger MF	Not conducted
*Panthera leo*	Andersen, K.F. and Vulpius, T. [21]	Urine	Solvent-based extraction	GC-MS	Not conducted	55 compounds found; several amines, aldehydes, ketones, alkenes, and dienes; acetone, 2-butanone, 1-pentene, 2-pentylfuran, heptanal, 1,2-cyclooctadiene and diethylbenzene potentially responsible for species identity	Not conducted
	Albone, E.S. and Gronnerberg, T.O. [23]	Anal sac secretions	Solvent-based extraction	GLC-MS, TLC	Not conducted	1-alkylglycerols and 2-hydroxy fatty acids, phenylacetic, 3-phenyl-propionic, and related hydroxylated acids were identified	Not conducted
	Soso, S.B. and Koziel, J.A., Manuscript in Review [26]	Marking fluid	SPME	mdGC-MS-O	mdGC-MS-O	81 volatile organic compounds comprise marking fluid; 19 volatile organic compounds were detected using olfactometry; 2,5-dimethyl-pyrazine, 3-methylcyclopentanone and 4-methylphenol responsible for characteristic odor of marking fluid	MF was analyzed in totality with urinous component and compared with previous literature analyzing the same content; 26 additional compounds were identified along with characteristic odorants
*Panthera leo persica*	Brahmachary, R.L. and Singh, M. [30]	Marking fluid	Solvent-based extraction	PC, TLC	Not conducted	Amines and free fatty acids are putative pheromones of MF; Minor differences between lipid composition of lion and tiger MF; Anal gland fluid is not found in MF	Not conducted
*Acinonyx jubatus*	Poddar-Sarkar, M., and Brahmachary, R.L. [8]	Marking fluid	Solvent-based extraction	GC-FID, TLC	Not conducted	C_2_-C_8_ free fatty acids	Not conducted
*Panthera pardus fusca*	Poddar-Sarkar, M. and Brahmachary, R.L. [24]	Marking Fluid	Solvent-based extraction	GC-FID	Not conducted	C_2_-C_9_ free fatty acids in the acidic fraction of steam distillate of marking fluid; Several amines were detected in the basic fraction of marking fluid; The amount of lipid extracted from MF is 1.15 mg/mL	Not conducted

Note: SEP = sample enrichment probe; SPME = solid phase microextraction; TLC = thin layer chromatography; GLC = gas liquid chromatography; GC-FID = gas chromatography-flame ionization detector; PC = paper chromatography; GC-MS = gas chromatography-mass spectrometry; mdGC-MS-O = multidimensional gas chromatography-mass spectrometry-olfactometry; and GC = gas chromatography.

**Table 2:** A list of all the VOCs in the marking fluid of *P. tigris altaica* identified using GC-MS-O. **Bolded entries** are compounds that are characteristic of the total aroma of tiger MF. Compounds were identified using spectral matches with the top five ions, odor descriptor matching, chemical standard confirmation (except for 2-AP), retention time, and the NIST library spectral matching.

No.	Compound Classification	RT (min)	CAS	Top 5 Ions and Relative Intensities (%)	R. Match Factor (%)	Aroma Descriptor by Panelist	Published Odor Descriptors	MOI (%)	PA	ODT (ppb)	SOAV
*Nitrogen containing compounds*											
1	2,5-Dimethyl-pyrazine ^a^	10.47	108-50-9	42(99),108(92),39(31),40(25),81(18)	80		Cocoa, Roasted Nuts, Roast Beef, Coffee ^b^		1.85 × 10^4^	8.00 × 10^2^–1.80 × 10^4^^c^	1.02 × 10^0^–2.31 × 10^1^
2	**2-Acetyl-1-pyrroline ^ᶲ^**	10.76	99583-29-6	43(99),41(54),42(24),83(13),39(11)	84	Basmati rice, Taco Shell, Nutty, Corn	Nutty, Popcorn, Toasted, Grain, Roasted, Basmati Rice, Malty ^b,d^	80	1.05 × 10^4^	0.10 × 10^0^^e^	1.05 × 10^5^
3	Indole ^a^	26.9	120-72-9	117(99),90(43),89(20),63(9),118(9)	96		Animal, Floral, Moth Ball, Fecal, Naphthelene ^b,f^		4.79 × 10^3^	1.40 × 10^2^^c^	3.42 × 10^1^
4	**Urea ^a^**	28.98	57-13-6	17(99),60(92),44(75),16(17),43(16)	96	Urinous, Ferret, Foul	Ammonia ^g^	30	8.35 × 10^3^		
*Ketones*											
5	Acetone ^a^	2.04	67-64-1	43(99),58(30),42(10),15(17),27(8)	96		Solvent, Ethereal, Apple, Pear ^b^		1.51 × 10^6^	5.00 × 10^5^^c^	3.02 × 10^0^
6	2-Butanone ^a^	2.56	78-93-3	43(99),73(32),29(18),57(10),27(8)	99		Acetone-like, Ethereal, Fruity, Camphor ^b^		6.98 × 10^5^	5.00 × 10^4^^c^	1.40 × 10^1^
7	3-Pentanone ^a^	3.62	96-22-0	43(99),57(54),44(35),86(32),41(27)	90	Body Odor, Plastic, Citrus, Bleach, Medicinal	Ethereal, Acetone ^b^	30	1.00 × 10^6^	7.00 × 10^4^^c^	1.43 × 10^1^
8	2,3-Butanedione ^a^	3.77	431-03-8	43(99),86(20),42(8),44(8),41(4)	93	Butter, Sweet, Cake Batter	Sweet, Buttery, Caramellic nuance ^b^	30	8.99 × 10^5^		
9	2-Methyl-3-pentanone ^a^	3.91	565-69-5	57(99),43(77),29(38),100(27),71(45)	90	Chemical	Mint ^b^	30	1.59 × 10^5^	5.00 × 10^3^^h^	3.18 × 10^1^
10	4-Heptanone ^a^	6.36	123-19-3	43(99),71(85),41(18),27(17),11(17)	92		Fruity, Cheese, Sweet, Cognac, Pineapple ^b^		5.54 × 10^5^	0.82 × 10^1^–4.10 × 10^1^^i^	1.35 × 10^4^
11	2-Heptanone ^a^	7.62	110-43-0	43(99),58(40),27(35),71(12),29(12)	95		Soapy, Fruity, Spicy, Sweet, Herbal, Coconut, Woody ^b^		1.26 × 10^6^	0.14 × 10^3^–3.00 × 10^3 c^	4.20 × 10^2^–9.02 × 10^3^
12	2-Nonanone ^a^	11.63	821-55-6	58(99),57(28),43(27),41(26),55(16)	81	Earthy, Grassy, Skunky, Foul, Onion, Rancid, Green pepper	Earthy, Herbaceous, Weedy, Green, Dirty ^b^	80	3.78 × 10^4^	0.05 × 10^2^–2.00 × 10^2^^c^	1.89 × 10^2^
13	2-Undecanone ^a^	15.22	112-12-9	58(99),43(58),59(32),71(29),41(18)	93		Waxy, Fruity, Creamy, Fatty, Orris Floral ^b^		2.90 × 10^4^	7 × 10^0^^c^	4.15 × 10^3^
*Amines*											
14	Trimethylamine ^a^	1.37	75-50-3	58(99),59(70),30(35),42(25),28(12)	95	Fish, Onion, Foul, Rancid, Skunky	Fishy, Oily, Rancid, Sweaty, Fruity ^b^	100	7.12 × 10^7^	3.70 × 10^−1^–10.60 × 10^−1^^c^	6.71 × 10^7^–1.92 × 10^8^
Aldehydes											
15	Hexanal ^a^	5.56	66-25-1	44(99),56(82),41(71),43(77),57(39)	83		Green ^b^		3.42 × 10^5^	4.50 × 10^−2^–5.00 × 10^−2^^c^	7.61 × 10^3^–6.84 × 10^4^
16	3-Methylbutanal ^a^	5.77	590-86-3	44(99),43 (86),41(49),57 (41),39(26)	95		Ethereal, Aldehydic, Chocolate, Peach, Fatty, Nutty ^b,d^		8.49 × 10^5^	0.20 × 10^0^–2.0 × 10^0^^c^	4.25 × 10^5^–4.25 × 10^6^
17	Nonanal ^a^	11.82	124-19-6	57(99),41(92),43(91),56(80),44(76)	88		Fatty, Floral-Rose, Waxy ^b,c^		1.52 x 10^4^	1.00 × 10^0^^c^	1.52 × 10^4^
18	**Furfural ^a^**	13.23	98-01-1	97(99),96(98),39(65),38(22),29(20)	97	Potato, Body odor, Earthy, Nutty	Sweet, Woody, Almond, Fragrant, Baked Bread ^b,f^	80	3.25 × 10^4^	3.00 × 10^3^–2.30 × 10^4^^c^	0.14 × 10^1^–1.10 × 10^1^
19	Benzaldehyde ^a^	14.04	100-52-7	106(99),77(97),105(97),107(80),39(63)		Fruit loops, Fruity, Sweet	Almond-like, Fruity, Cherry, Sweet, Bitter, Sharp ^b^	100	1.75 x 10^5^	3.50 × 10^2^–3.50 × 10^3^^c^	5.00 × 10^1^–5.00 × 10^2^
*Alcohols*											
20	Ethanol ^a^	3.02	64-17-5	31(99),45(55),29(32),27(24),46(21)	97		Strong, Alcoholic, Ethereal, Medical ^b^		1.58 × 10^6^	1.00 × 10^5^^c^	1.58 × 10^1^
21	1-Butanol ^a^	7.16	71-36-3	56(99),31(98),41(90),43(70),27(58)	95		Medicine, Fruit, Wine ^f^		4.43 × 10^5^	5.00 × 10^2^^c^	8.86 × 10^2^
22	3-Methyl-1-butanol ^a^	8.77	123-51-3	55(99),42(90),41(82),43(84),70(73)	96		Fusel, Alcoholic, Pungent, Etherial, Cognac, Fruity, Banana and Molasses ^b^		9.37 × 10^4^	2.50 × 10^2^-3.00 × 10^2^^c^	3.12 × 10^2^–3.74 × 10^2^
23	1-Hexanol ^a^	11.13	111-27-3	56(99),43(83),41(59),55(58),42(57)	86		Pungent, Etherial, Fusel Oil, Fruity and Alcoholic, Sweet with a Green Top Note ^b^		7.36 × 10^3^	2.50 × 10^3 c^	2.94 × 10^0^
24	1-Octanol ^a^	14.76	111-87-5	56(99),55(88),41(81),73(75),70(61)	80	Roasted, Earthy, Grassy, Green Pepper	Waxy, Green, Orange, Aldehydic, Rose, Mushroom ^b^	30	2.78 × 10^4^	1.10 × 10^2 c^	2.53 × 10^2^
25	Benzyl Alcohol ^a^	19.68	100-51-6	79(99),77(57),108(90),107(70),51(22)	92		Floral, Rose, Phenolic, Balsamic ^b^		1.94 × 10^4^	1.00 × 10^4^^c^	1.94 × 10^0^
26	Phenylethyl alcohol ^a^	20.16	60-12-8	91(99),51(64),39(75),92(60),77(48)	91	Citrus, Sweet	Rose, Floral ^b^	30	9.04 × 10^4^	7.50 × 10^2^–1.10 × 10^3 c^	8.22 × 10^1^
*Sulfur containing compounds*											
27	Dimethyl disulfide ^a^	5.39	75-18-3	94(99),79(58),45(50),46(25),47(20)	97		Sulfury, Onion, Sweet, Corn, Vegetable, Cabbage, Tomato, Green, Radish ^j^		4.71 × 10^5^	2.00 × 10^0^–1.20 × 10^0^^c^	3.93 × 10^4^–2.94 × 10^6^
28	Dimethyl trisulfide ^a^	11.47	3658-80-8	126(99),79(56),45(33),47(23),111(18)	92	Onion, Skunky	Foul, Sulfur, Fish, Cabbage ^f^	60	1.08 × 10^4^	0.50 × 10^−2^–1.00 × 10^−2^^c^	1.08 × 10^6^
*Acids*											
29	Valeric acid ^a^	17.6	109-52-4	60(99),73(37),41(15),29(14),27(12)	98	Rancid, Foul, Unknown	Rancid, Sickening, Putrid, Acidic, Sweaty, Sour, Cheese-like ^b^	30	8.90 × 10^3^	3.00 × 10^3^^c^	2.97 × 10^0^
30	Octanoic acid ^a^	22.53	124-07-2	60(99),73(62),43(42),41(39),55(37)	93		Fatty, Waxy, Rancid Oily, Vegetable, Cheesy ^b^		1.55 × 104	3.00 × 10^2 c^	5.18 x 100
*Amides*											
31	Acetamide ^a^	17.94	60-35-5	59(99),44(89),43(60,42(29),18(27)	98		Mousy ^b^		1.87 × 10^5^		
*Phenols*											
32	4-Methylphenol ^a^	22.6	106-44-5	107(99),108(85),77(32),79(21),51(16)	97	Barnyard, Chemical, Animal, Earthy	Phenolic, Narcissus, Animal, Mimosa ^b^	80	2.97 × 10^3^	5.50 × 10^1^^c^	5.40 × 10^1^
33	Phenol ^a^	21.54	108-95-2	93(99),66(39),65(28),39(25),40(15)	97		Phenolic, Plastic, Rubber ^b^		2.12 × 10^4^	5.9 × 10^3^^c^	3.60 × 10^0^

Abbreviations: CAR/PDMS—Carboxen polydimethylsiloxane; DVB/CAR/PDMS—divinylbenzene/Carboxen polydimethylsiloxane; PDMS—polydimethylsiloxane; polydimethylsiloxane/divinylbenzene; GC—gas chromatography; RT—retention time; CAS—Chemical Abstracts Service Numbers; RA-relative abundance; SOAV—surrogate odor activity value; MOC—Measured Odor characters; MOI—measured odor intensity; ODT—Odor detection threshold; bolded entries are compounds that are characteristic of the total aroma of tiger MF; 2-AP—2-acetyl-1-pyrroline—is placed in Table 2 because it was implicated as a characteristic odorant in Bengal tiger MF [4,5,32]; **^ᶲ^** Compounds verified with spectral matches with the top five ions, odor panelists’ detection, and published odor descriptors; ^a^ Compounds verified with retention time and ion confirmation of standards (except for 2-AP); ^b^ Good Scents Company [55]; ^c^ Leffingwell & Associates [56]; ^d^ Flavornet [57]; ^e^ Flavor Chemistry and Odor Thresholds [58]; ^f^ Encyclopedia Britannica [59]; ^g^ Urea (Ultra-Pure Grade) Safety Data Sheet [60]; ^h^ Measurement of Odor Threshold by Triangle Odor Bag Method [61]; ^i^ Fenaroli’s Handbook of Flavor Ingredients 5th Edition [62]; ^j^ Flinn Scientific, Inc. Safety Data Sheet (SDS) [63]; ^k^ Haz-Map [64].

**Table 3:** Experimental treatments and the different fiber types used in the experimental design.

	Treatments			
Fiber Type	Sample Size	Temperature	Time	Sample Agitation
**85 µm CPDMS**	0.25 mL	25 °C	1 h	None
	0.50 mL		24 h	0.20 cm × 0.50 cm Stir bar @ 1000 rpm
	0.25 mL	37 °C	1 h	None
	0.50 mL		24 h	0.20 cm × 0.50 cm Stir bar @ 1000 rpm
**75 µm CPDMS**	0.25 mL	25 °C	1 h	None
	0.50 mL		24 h	0.20 cm × 0.50 cm Stir bar @ 1000 rpm
	0.25 mL	37 °C	1 h	None
	0.50 mL		24 h	0.20 cm × 0.50 cm Stir bar @ 1000 rpm
**50/30 µm DVB/CPDMS**	0.25 mL	25 °C	1 h	None
	0.50 mL		24 h	0.20 cm × 0.50 cm Stir bar @ 1000 rpm
	0.25 mL	37 °C	1 hour	None
	0.50 mL		24 h	0.20 cm × 0.50 cm Stir bar @ 1000 rpm
**100 µm PDMS**	0.25 mL	25 °C	1 h	None
	0.50 mL		24 h	0.20 cm × 0.50 cm Stir bar @ 1000 rpm
	0.25 mL	37 °C	1 h	None
	0.50 mL		24 h	0.20 cm × 0.50 cm Stir bar @ 1000 rpm
**65 µm PDMS/DVB**	0.25 mL	25 °C	1 h	None
	0.50 mL		24 h	0.20 cm × 0.50 cm Stir bar @ 1000 rpm
	0.25 mL	37 °C	1 h	None
	0.50 mL		24 h	0.20 cm × 0.50 cm Stir bar @ 1000 rpm

Abbreviations: CAR/PDMS = Carboxen polydimethylsiloxane; DVB/CAR/PDMS = divinylbenzene/ Carboxen polydimethylsiloxane; PDMS = polydimethylsiloxane; PDMS/DVB = polydimethyl-siloxane/divinylbenzene.

## Supplementary Materials

Supplementary materials can be accessed at: http://www.mdpi.com/1420-3049/21/7/834/s1.
