# Supplementary material for: Analysis of Odorants in Marking Fluid of Siberian Tiger (Panthera tigris altaica) Using Simultaneous Sensory and Chemical Analysis with Headspace Solid-Phase Microextraction and Multidimensional Gas Chromatography-Mass Spectrometry-Olfactometry
Source: Molecules. 2016 Jun 25;21(7):834. doi: 10.3390/molecules21070834 (PMC6273184; doi:10.3390/molecules21070834)
Supplement: Supplementary file 1 [file molecules-21-00834-s001.pdf]

Supplementary Materials: Analysis of Odorants in Marking Fluid of Siberian Tiger (*Panthera tigris altaica*) Using Simultaneous Sensory and Chemical Analysis with Headspace Solid-Phase Microextraction and Multidimensional Gas Chromatography-Mass Spectrometry-Olfactometry

Simone B. Soso and Jacek A. Koziel

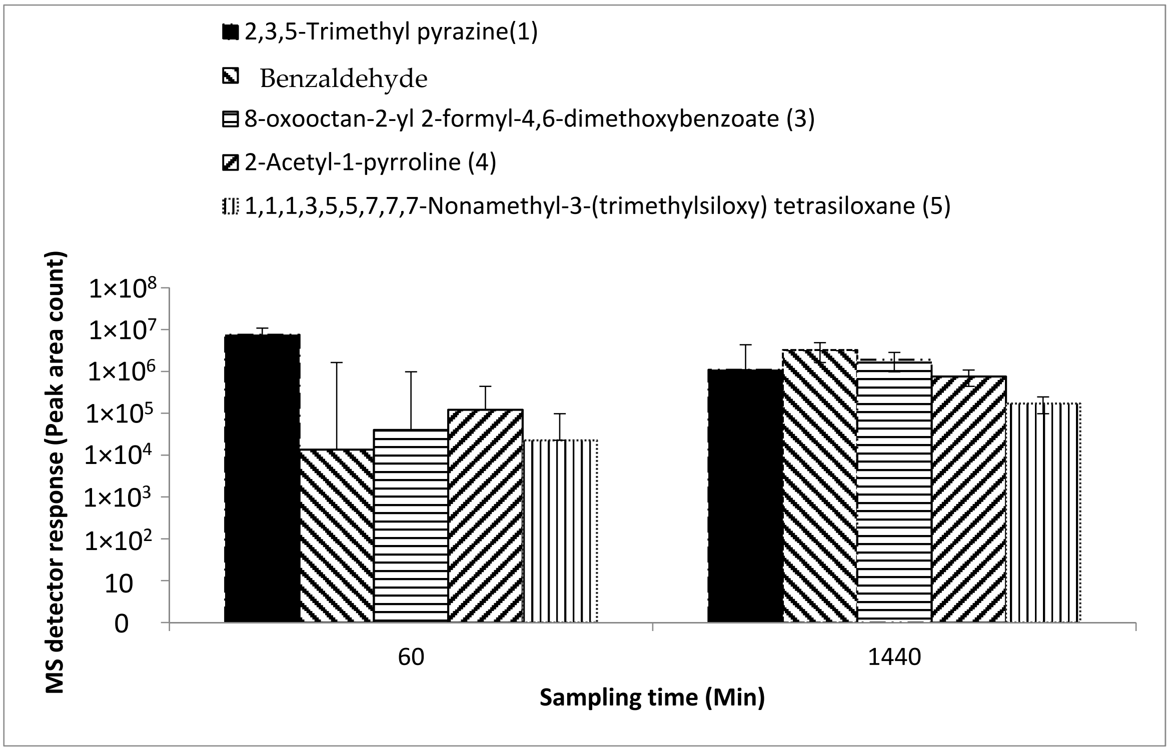

Figure S1. Effects of SPME extraction time for five odorous compounds released from the marking fluid of *P. tigris altaica* with a 75 μm CAR/PDMS fiber. Extraction time = 60 min, and 1440 min (24 h). Error bars show the standard deviation of the mean (n = 3). Marking fluid (0.25 mL) and a stir bar were inserted into a 2 mL glass vial with a PTFE coated septa for a period of 30 min for equilibration. These compounds were confirmed with the top five ions, odor descriptors observed by panelist not chemical standards.

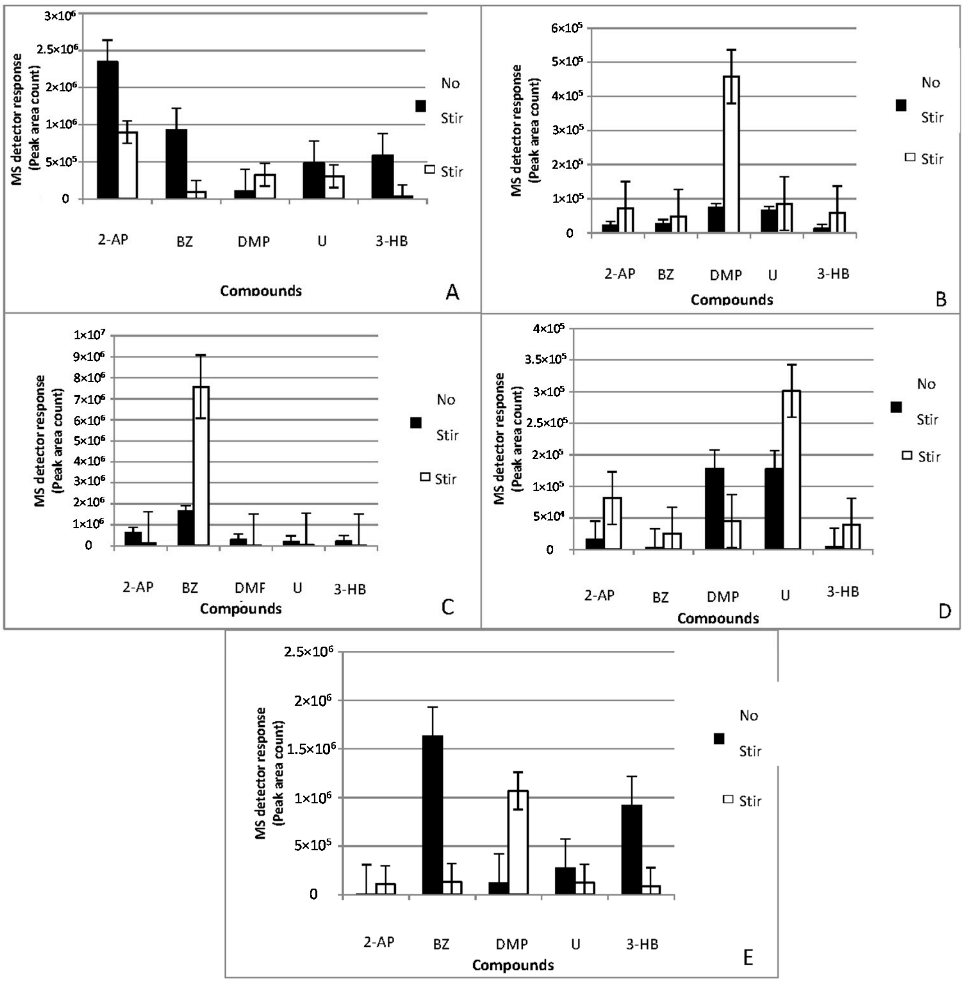

Figure S2. Effects of agitation 1h extraction, temperature 37 °C, with a 0.25 mL sample. (**A**) 75 μm CAR/PDMS SPME fiber; (**B**) 50/30 μm DVB/Carboxen/PDMS SPME fiber; (**C**) 100 μm PDMS SPME fiber (**D**) 65 μm DVB/Carboxen/PDMS SPME fiber; and (**E**) 85 μm CAR/PDMS SPME fiber. These compounds were confirmed with the top five ions, odor descriptors observed by panelist not chemical standards. Abbreviations: U = Urea, 2-AP=2-Acetyl-1-pyrroline, 3-HB = 3-Hydroxybutanal, DMP = 2,5-Dimethyl-pyrazine, BZ = Benzaldehyde.

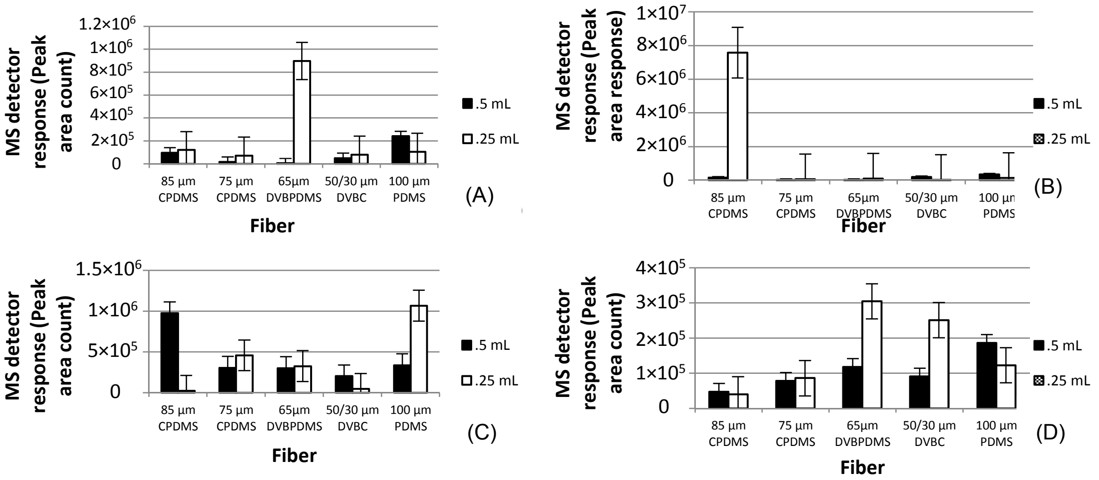

Figure S3. Effects of sample quantity (0.50 mL and 0.25 mL) for the identification of (**A**) 2-Acetyl-1-pyrroline; (**B**) 3-HB = 3-Hydroxybutanal; (**C**) 2,5-Dimethyl-pyrazine; and (**D**) Benzaldehyde, key characteristic odor compounds, released from the marking fluid of *P. tigiris altaica*. These compounds were tentatively confirmed with the top five ions and odor descriptors observed by panelist.

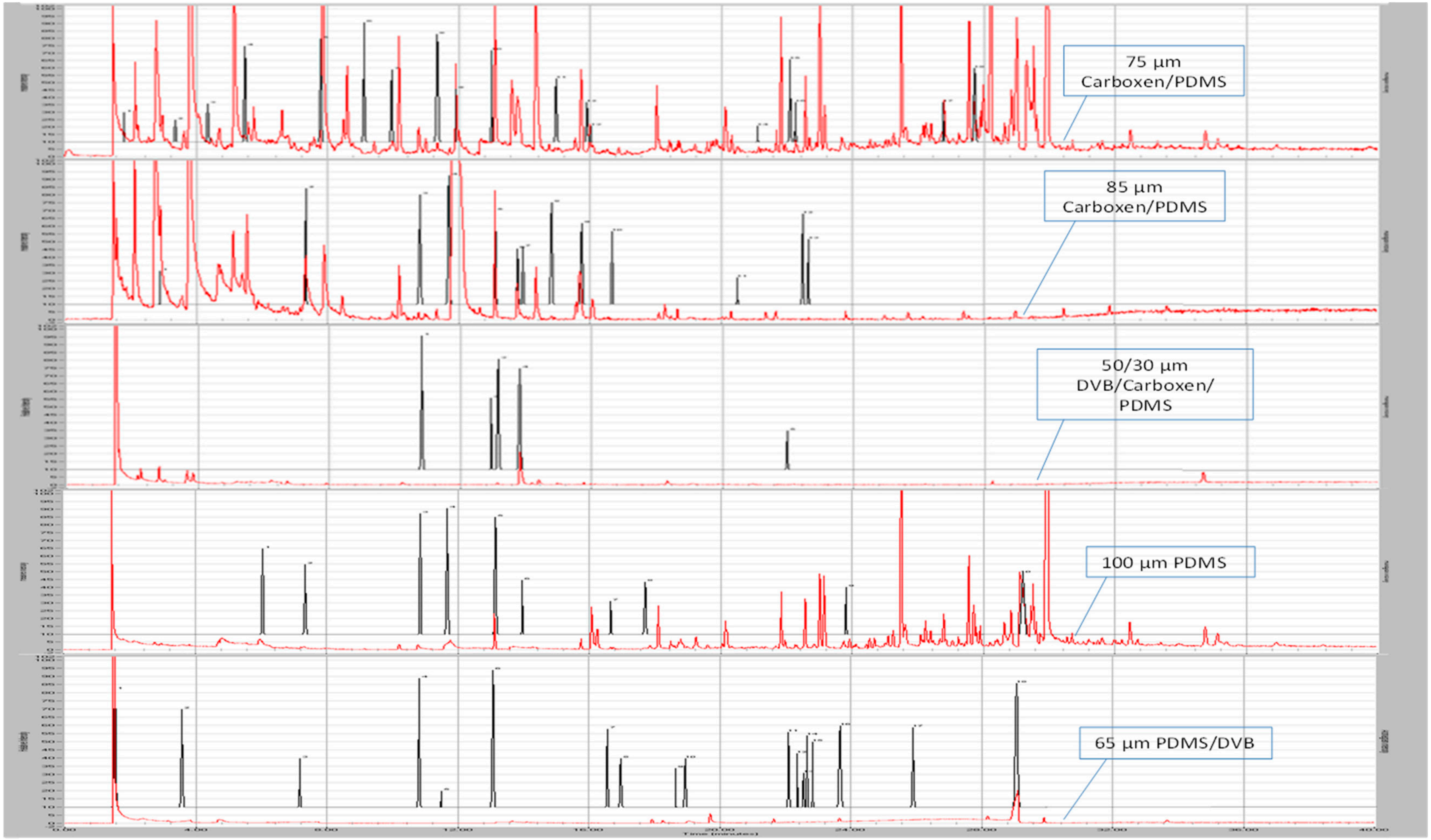

Figure S4. Comparison of aromagrams and total ion chromatograms of *P. tigris altaica* marking fluid headspace extracted with 85 µm Carboxen/PDMS, 50/30 µm DVB/Carboxen/PDMS, 100 µm PDMS, 65 µm PDMS/DVB coating, and 75 µm Carboxen/PDMS SPME fibers and analyzed using GC-MS-Olfactometry. Extraction time = 24 h ($n = 3$). Temperature = 37 °C. Samples were analyzed in MS scan mode: total ion scan. Extractions with 75 µm Carboxen/PDMS SPME fiber were associated with the largest number of odorous compounds (14) with a total of 32 odorous events.

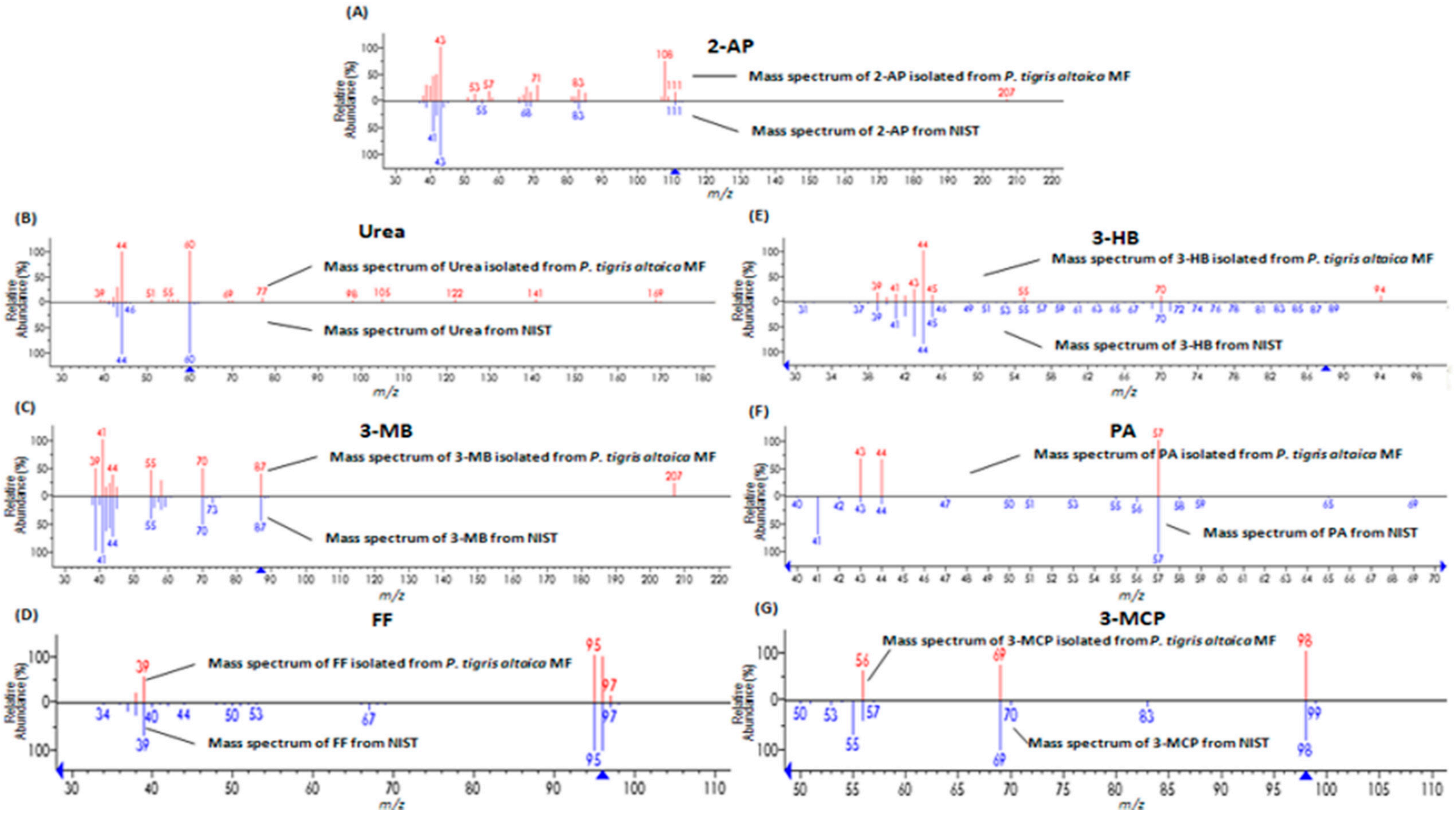

Figure S5. Identity confirmation of seven compounds responsible for characteristic tiger marking fluid odor through NIST mass spectral library matching ((*R*)-3-methylbutanamine, 3-hydroxy-butanal, propanedionic acid, and (*R*)-3-methylcyclopentanone) and chemical standard confirmation (urea, furfural, and 2-acetyl-1-pyrroline). All of the characteristic compounds were selected based on their 'nutty', 'urinous', and/or 'corn-like' aromas. For those compounds not identified with chemical standards, odor panelist and published odor descriptors aided in their suggested identification. (**A**) 2-Acetyl-1-pyrroline; (**B**) urea; (**C**) 3-methylbutanamine; (**D**) furfural; (**E**) 3-hydroxybutanal; (**F**) propanedioic acid; and (**G**) (*R*)-3-methylcyclopentanone. Abbreviations: 2-AP = 2-Acetyl-1-pyrroline, 3-MB = (*R*)-3-MethyIbutanamine, 3-HB = 3-Hydroxy-butanal, FF = Furfural, PA = Propanedioic Acid, 3-MCP (R)-3-Methylcyclopentanone, m/z = mass-to-charge.

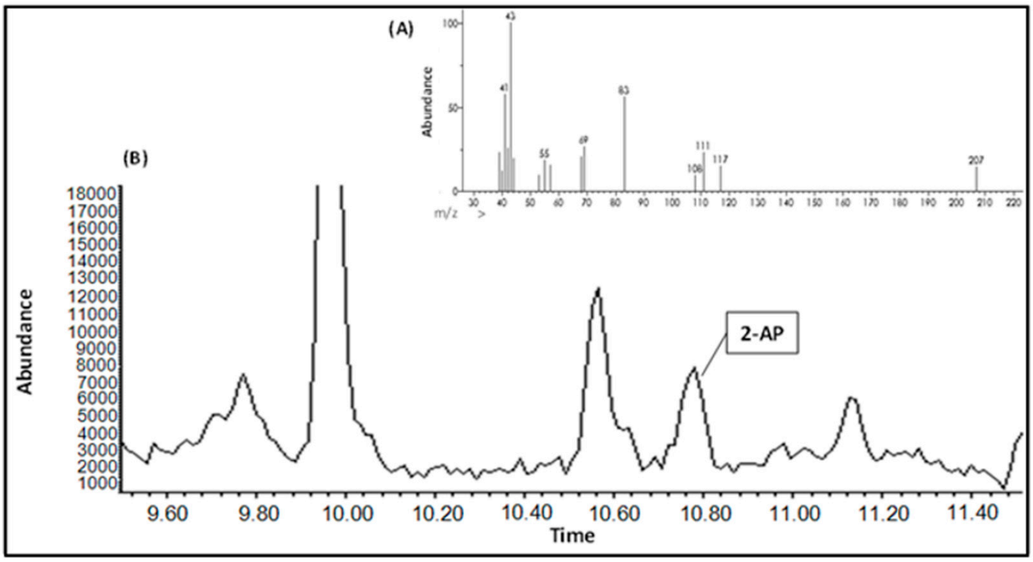

Figure S6. (**a**) The mass spectrum of 2-AP peak is shown in the upper right comer; (**b**) The mass spectrum of 2- AP isolated from volatiles collected from the headspace of *P. tigris altaica* marking fluid.

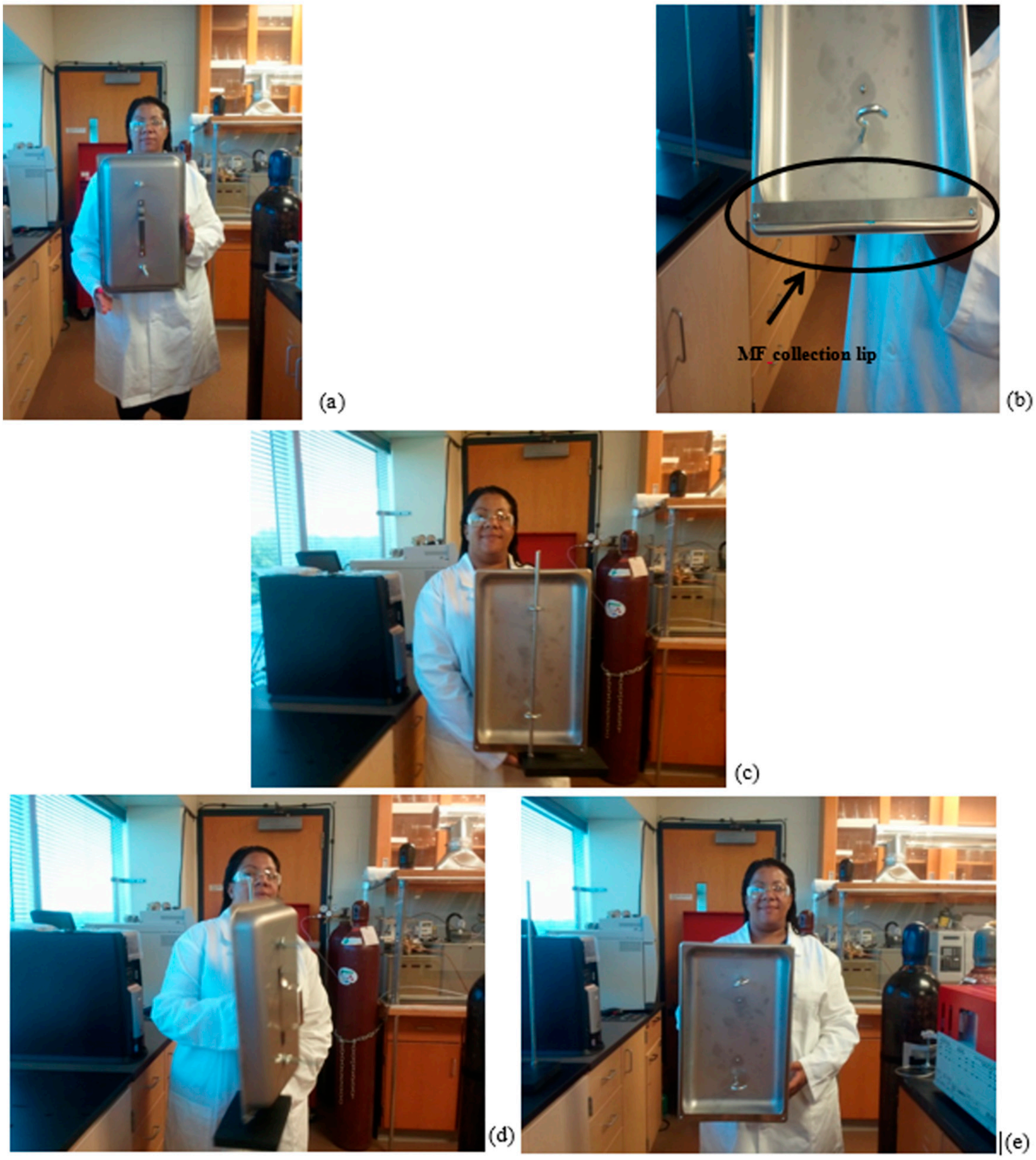

Figure S7. Prototype of the tiger marking fluid collection system that was attached to the cage of the indoor tiger enclosure areas. (**a**) Exterior portion of the collection device; (**b**) the lip at the base of the collection device that the marking fluid will drain into (**c**) representative of the placement of the collection system on the case bars; (**d**) side profile of the collection device; (**e**) interior area of the collection device.

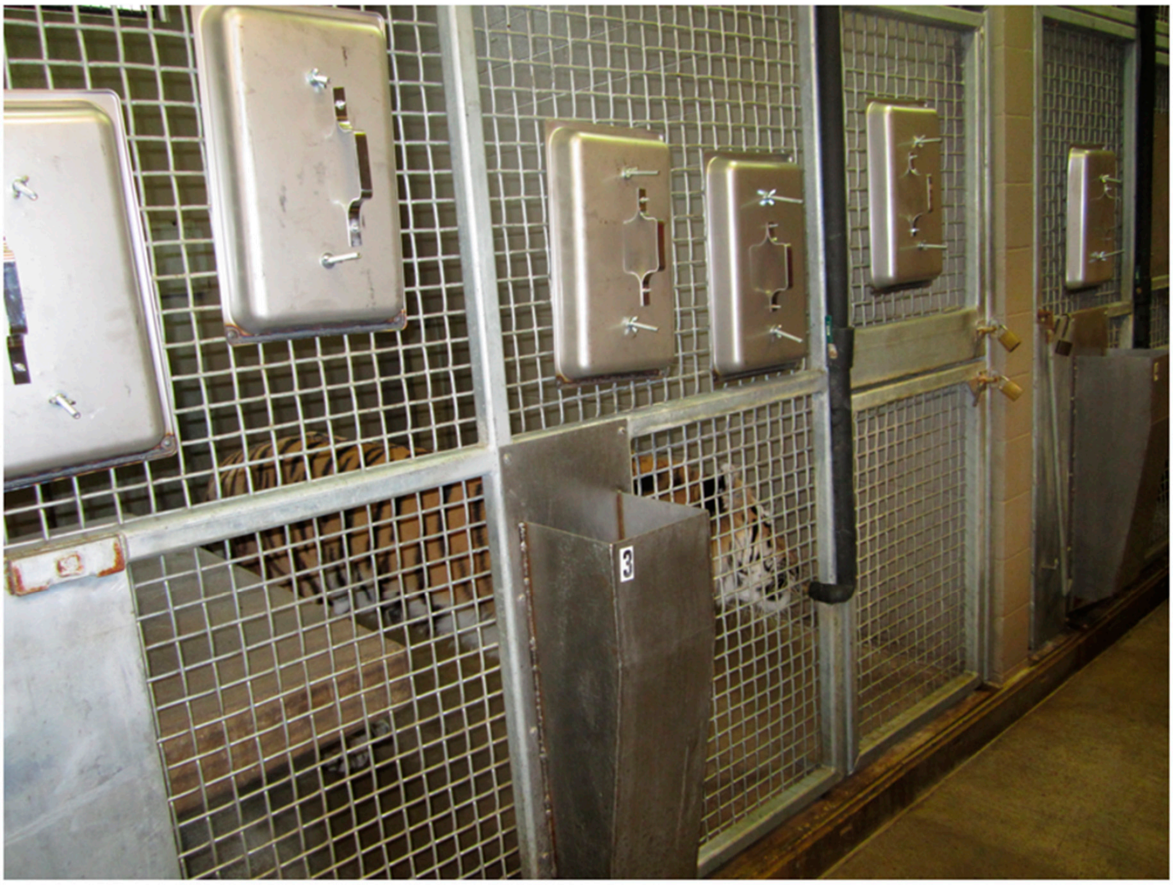

Figure S8. Placement of the tiger marking fluid collection system attached to the cage of the indoor tiger enclosure areas.

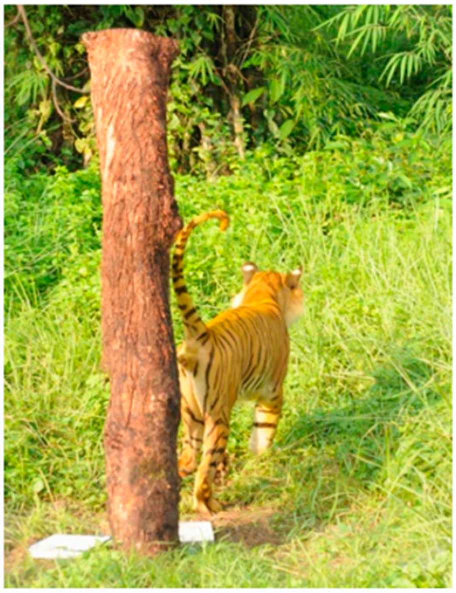

Figure S9. A *Panthera tigris* performing scent-marking behaviors in its outdoor enclosure releasing marking fluid.

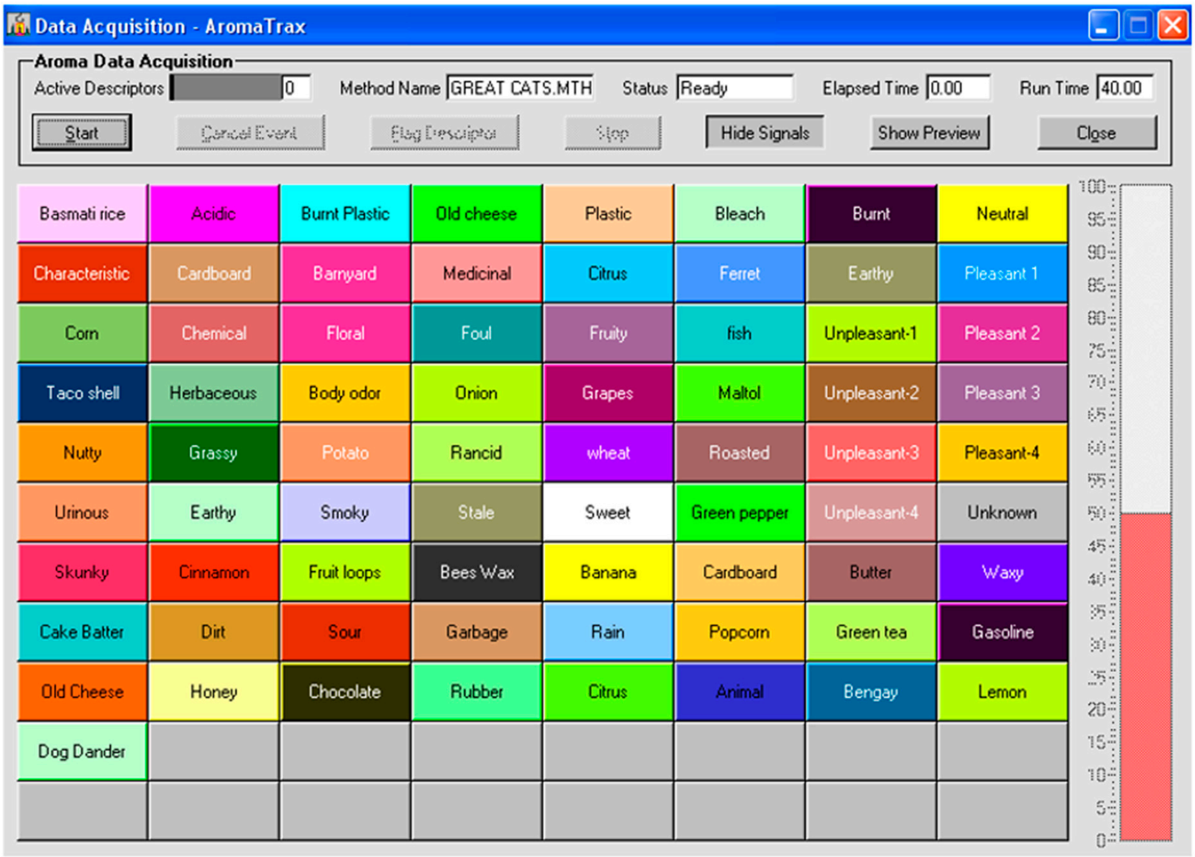

Figure S10. Odor descriptor panel used to characterize the odorous compounds within the tiger markings.

Table S1. Summary of all unconfirmed peaks in the chromatogram of *P. tigris altaica* MF. Compounds were listed by identifying markers: spectral matches with the top five ions, odor descriptors observed by panelist, and retention time. **Bolded entries** are unconfirmed compounds (3-methylbutanamine-RT = 7.25 min, (*R*)-3-methylcyclopentanone-RT = 8.24 min, propanedioic acid-RT = 13.81 min, and 3-hydroxybutanal-RT = 5.89 min) that are characteristic odorants of the total aroma of Siberian tiger MF.

No.	RT (min)	Top 5 Ions and Relative Intensities (%)	Aroma Descriptor by Panelist	Measured Odor Intensity (%)
1	4.85	71(99),43(88),55(68),41 (40),39(30)	Foul, Onion	30
**2**	**5.89**	**44(99),43(96),41(92),58(82),29(48)**	**Body Odor, Plastic, Urinous, Skunky**	**80**
3	5.98	98(99),97(92),71(87),41 (55),67(50)	Body Odor, Sour, Skunky	30
4	6.45	267(99),269(96),126(63,195(43),282(37)	Plastic, Smoky	100
5	6.64	43(99),86(20)41(16),58(16),71(16)		
6	6.78	81(99),80(75),53(28),42(23),39(22)	Foul, Sour	15
7	7.07	43(99),57(71),41(41),71 (38),85(29)	Grassy, Earthy	80
**8**	**7.25**	**29(99),44(81),43(66),18 (40),41(32)**	**Skunky, Urinous**	**30**
9	7.48	29(99),44(65),15(64),14(28),43(18)		
**10**	**8.24**	**69(99),55(63),42(62),98(62),41(45)**	**Urinous, Foul**	**60**
11	8.99	42(99),55(94),41(68),70(48),31(36)	Foul	15
12	9.76	55(99),42(57),98(56),41(20),69(21)		
13	11.21	121(99),79(23),120(20),106(15),39(5)	Earthy, Grassy, Herbaceous	60
14	11.98	42(99),122(66),39(17),81(16),40(9)	Herbaceous, Grassy, Earthy, Skunky, Foul, Onion	60
15	12.80	73(99),281(86),147(60),415 (34),327(33)	Sweet, Fruity	30
16	13.56	57(99),41(45),55(36),43(28),56(27)	Body Odor, Plastic, Potato, Earthy	30
**17**	**13.81**	**42(99),45(86),60(77),44 (62),43(62)**	**Skunky, Foul, Urinous, Body Odor**	**30**
18	14.90	57(99),43(72),71(58),85(55),41(28)	Onion, Sulfur	80
19	16.21	30(99),91(15),92(12),121(5),65(10)		
20	16.58	150(99),107(75),108(70),43(54),42(52)		
21	16.65	42(99),28(65),41(40),29(49),27(20)		
22	16.95	136(99),54(90)	Stale, Sweet, Medicinal, Foul	60
23	18.67	118(99),91(73),119(41),104(31),132(28)		
24	18.91	207(99),133(51),191(85),177(27),193(25)	Body odor, Smoky, Unknown	60
25	20.68	57(99),71(91),43(78),85(48),41(43)		
26	20.93	41(99),43(90),29(50),55(50),57(48)	Waxy, Sweet	15
27	21.01	94(99),109(78),66(54),39(34),43(12)		
28	21.52	170(99),51(52),77(40),141(40),39(30)		
29	21.72	94(99),66(70),39(62),65(50),96(18)		
30	22.36	43(99),41(95),39(35),69(35),15(30)		
31	22.83	41(99),55(49),83(48),110(47),43(42)		
32	23.62	121(99),149(58),138(20),196(20)		
33	24.19	120(99),135(65),92(54),65(17),43(9)		
34	24.23	120(99),135(45),92(40),65(10),39(2)	Sweet, Fruity, Grape	30
35	24.49	43(99),58(59),85(25),59(27),41(20)	Sweet, Fruity	15
36	25.86	55(99),70(77),41(61),43(61),29(30)		

37	26.16	43(99),41(90),55(85),57(84),69(59)		
38	28.34	30(99),91(36),43(30),61(20)40(10)		
39	28.38	91(99),92(33),195(24),194(20),65(10)		
40	28.70	105(99),122(95),77(75),51(50),106(15)		
41	28.74	170(99),169(81),141(53),142(18),115(55)		
42	28.91	31(99),32(20),30(10),29(42),60(35)	Fruity, Grape, Sweet, Waxy	30
43	29.22	105(99),77(45),51(15),106(5),50(5)		
44	29.39	91(99),136(56),92(30),65(15),39(8)		
45	29.53	150(99),44(58),166(40),50(10),104(8)		
46	29.99	73(99),60(95),43(76),41(50),57(70)		
47	30.88	44(99),45(60),29(22),52(12),15(6)		
48	33.79	69(99),81(55),41(2),136(25),137(24)		

Abbreviations: No-Number; RT-Retention Time.
